# Using Biological Pathway Data with Paxtools

**DOI:** 10.1371/journal.pcbi.1003194

**Published:** 2013-09-19

**Authors:** Emek Demir, Özgün Babur, Igor Rodchenkov, Bülent Arman Aksoy, Ken I. Fukuda, Benjamin Gross, Onur Selçuk Sümer, Gary D. Bader, Chris Sander

**Affiliations:** 1 Computational Biology Center, Memorial Sloan-Kettering Cancer Center, New York, New York, United States of America; 2 The Donnelly Centre, University of Toronto, Toronto, Ontario, Canada; 3 Tri-Institutional Training Program, Computational Biology and Medicine New York, New York, United States of America; 4 Intelligent Information Infrastructure Division, National Institute of Advanced Industrial Science and Technology, Tsukuba, Japan; UCSD, United States of America

## Abstract

A rapidly growing corpus of formal, computable pathway information can be used to answer important biological questions including finding non-trivial connections between cellular processes, identifying significantly altered portions of the cellular network in a disease state and building predictive models that can be used for precision medicine. Due to its complexity and fragmented nature, however, working with pathway data is still difficult. We present Paxtools, a Java library that contains algorithms, software components and converters for biological pathways represented in the standard BioPAX language. Paxtools allows scientists to focus on their scientific problem by removing technical barriers to access and analyse pathway information. Paxtools can run on any platform that has a Java Runtime Environment and was tested on most modern operating systems. Paxtools is open source and is available under the Lesser GNU public license (LGPL), which allows users to freely use the code in their software systems with a requirement for attribution. Source code for the current release (4.2.0) can be found in Software S1. A detailed manual for obtaining and using Paxtools can be found in Protocol S1. The latest sources and release bundles can be obtained from biopax.org/paxtools.

This is a *PLOS Computational Biology* Software Article

## Introduction

The total volume of computational pathway data mapped by biologists has entered a rapid growth phase. There are more than 100,000 biochemical reactions and more than a million molecular interactions in publicly available 325 on-line resources [1]. Pathway analysis has been used to determine genetically altered pathways in diverse cancer types [2], [3], predict the functions and phenotypic effects of previously uncharacterized cancer genes [4], [5], prioritize candidate genes and pathways for functional validation in model systems [6], [7], identify network biomarkers correlated with clinical outcome in human disease [5], and translate genomic information into new targeted clinical applications [8].

Despite these promising studies, the use of computable pathway information in biology is still in its infancy. Pathway data sources were originally developed to use their internal pathway representation, resulting in a heterogeneous set of resources that are difficult to combine and use. There are two key technical challenges we need to address to streamline the use of this rapidly expanding information : (i) standardizing and integrating pathway data from multiple heterogeneous sources and (ii) developing methods and tools that can help scientists easily access, map and analyze pathway information.

BioPAX (Biological Pathway Exchange) [9] a standard language developed by a community of pathway data providers, tool developers and scientists enables data exchange between data providers and acts as a common interface for software tools to access pathway data from multiple sources. Recently released BioPAX Level 3 covers a large spectrum of pathway types, including signaling and metabolic pathways as well as genetic and protein-protein interactions. BioPAX, as a common discussion forum, also led to convergence on the semantics of pathway representation over the years. There are multiple aggregation services for publicly available pathway data in BioPAX [10], [11]. Pathway Commons (PC) [12], for example, validates, normalizes and integrates pathway information from Reactome [13], NCI/Nature PID [14], BioCyc [15], Panther Pathway [16], PhosphoSitePlus [17] and other major pathway databases.

The second key challenge is to build methods, algorithms and software tools to work with pathway data to answer biological questions [18]–[21]. Given the large number of classes and properties in BioPAX, developing software that consumes BioPAX can be a daunting task. To address this problem, we developed Paxtools, an open-source, Java library that contains facilities and algorithms for common but difficult to implement tasks such as reading, writing, searching, merging, comparing and transforming pathway information. Paxtools is the only complete BioPAX API available and is quickly becoming a common platform for accessing and analyzing pathway data as well as development of pathway software [22]–[25].

## Design and Implementation

At the core of Paxtools is a complete and consistent object-oriented implementation of the BioPAX specification. A BioPAX record includes pathways, interactions, reactions and their participants. These elements are implemented as Java beans that have methods to access and manipulate the properties as described in BioPAX specification. The Model class acts as a container that allows adding, removing and searching for elements.

BioPAX uses OWL semantics that are not automatically covered by object oriented languages such as Java. Properties can be symmetric, transitive or subtyped into other properties. For example, a protein binding relationship is symmetric, if A binds to B, the inverse is also true. Also, the “standard name” property is a subtype of “name” property, so updating the standard name of the protein should also update its list of names. Paxtools implements these additional semantics and automatically updates object fields.

In the BioPAX specification, properties are unidirectional for brevity. For example, the “participant” property links interactions to physical entities. Paxtools provides additional “inverse” links for key properties that allow efficient bidirectional navigation. These inverse links are also automatically updated when the forward link is updated.

Paxtools includes two input/output handlers for reading and writing BioPAX files. Jena based IO can handle most OWL encodings but can be relatively slow and demands a significant amount of memory for very large files. The StaX based “Simple” IO handler class can only read BioPAX formatted as RDF/XML and does not have the flexibility of the Jena handler but it can read hundreds of thousands of elements within minutes with a very small memory footprint.

**Figure 1:**
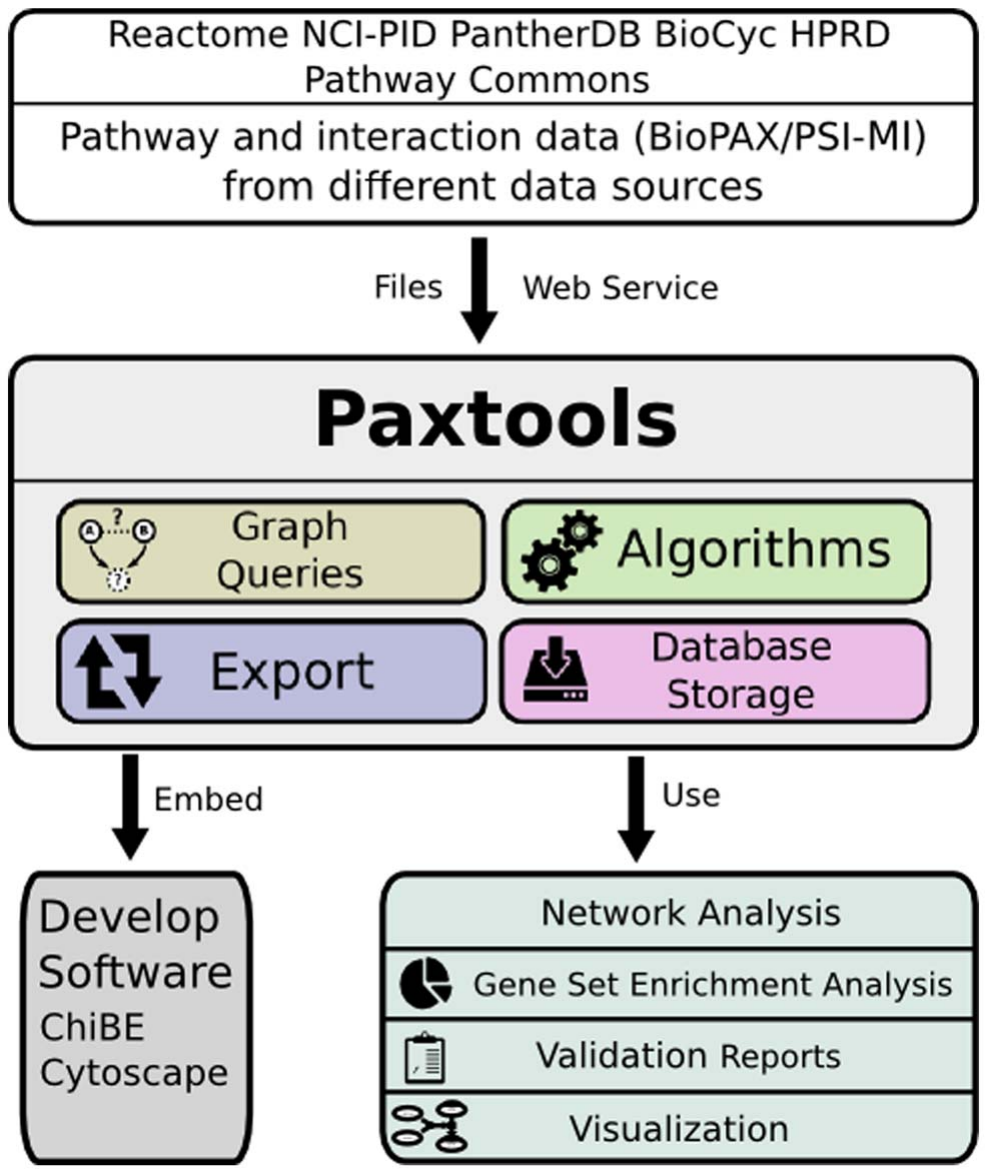
Paxtools can be used to obtain pathway data from different sources and use the data to answer a wide range of biological questions. Paxtools facilitates working with pathway information by removing technical barriers and acts as a common development platform for other tools and algorithms.

On top of this foundation Paxtools implements methods, algorithms and tools to solve common tasks encountered while working with pathway data (Figure 1). Some components, such as graph search algorithms, are difficult to implement correctly without a detailed understanding of BioPAX. For example, developers unfamiliar with the BioPAX specification might not be aware of generic entities and their semantics and fail to traverse these portions of the network. These algorithms in return might fail to find interesting and important interactions. Paxtools components were developed by researchers who have extensive experience working with BioPAX and are involved in BioPAX development. They were further tested and corrected by an active user community. Some of these components include:

### Efficient traversal and editing

The “Controller” package allows users to manipulate BioPAX models without actually hard coding property and class names, define XML Path Language-like queries and traverse the object graph efficiently. An example which implements an object inspector to display the properties of objects can be found in the user's guide (Text S1). This pattern considerably simplifies development of BioPAX exporters and other tools and makes it easier to extend and update them to support future changes in the BioPAX specification.

### Graph search algorithms for biological questions

**Figure 2:**
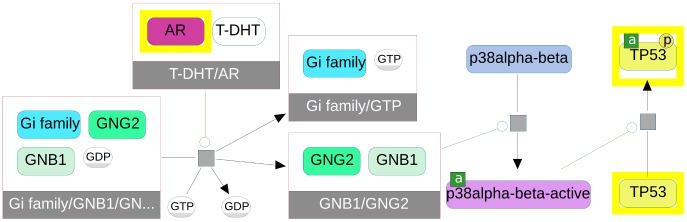
A plausible signaling path (yellow) between the androgen receptor (AR, top) and the TP53 protein (p53, right) is found by a Paxtools “paths-between” query and visualized in the Chisio BioPAX Editor. These cross-talks are difficult to find manually without a graph search because of the large number of connections to/from AR and p53 and because of fragmentation of data across multiple pathway data sources.

Paxtools offers an array of graph search algorithms that are specifically targeted towards answering common biological questions. For example a mutually exclusive relationship between the mutations observed in two genes in a set of drug-resistant tumors can be explained by a common downstream pathway. Similarly, an observed correlation in gene expression between two genes can be caused by a signaling path that connects one gene to the control of the expression of the other. Starting from a set of entities, users can find common upstream and downstream events, feedback loops, connecting paths and networks of interest (Figure 2). A list of algorithms and their complexity analysis can be found in [26].

### Algorithms for merging, extracting and reducing pathways

**Figure 3:**
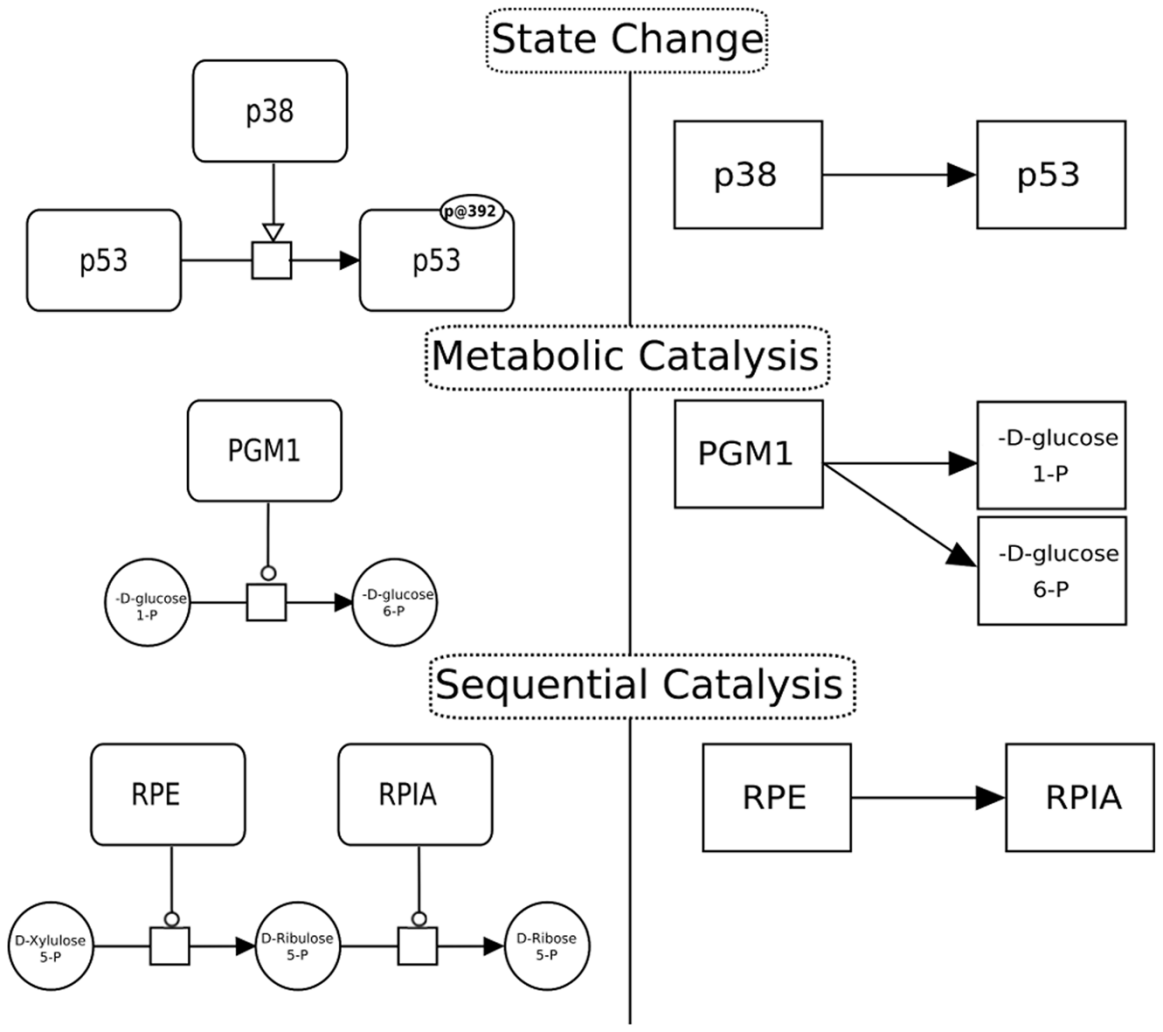
A Sif (Simple Interaction Format) rule reduces mechanistic BioPAX interactions (left column) to simpler binary interactions (right column) based on the pattern defined in the rule. Different rules should be used dependent on the biological question at hand. For example a State Change interaction is inferred when the rule detects phosphorylation of p53 by p38. This is a useful relationship that can be applied to protein signaling cascade analysis from proteomics data. “Sequential Catalysis” rule on the other hand links entities that are responsible for catalyzing subsequent reactions. This is a relationship that frequently occurs in metabolic pathways and is useful for metabolomic studies where changes in the concentration of substrates are observed.

Working with BioPAX often requires complex manipulations to merge models from different sources, extract subparts or reduce them to simpler formats (Figure 3). Paxtools provides an array of carefully designed algorithms for these tasks that pays considerable attention to the subtle details of the BioPAX format such as subsumption relationships between entities (i.e. generic proteins and complex molecules).

### Converting to and from different formats

Paxtools can convert Proteomics Standards Initiative - Molecular Interaction (PSI-MI) [27] models to BioPAX Level 3. In addition, BioPAX models can be exported back to OWL and several other useful formats, including Systems Biology Graph Notation markup language (SBGN-ML) [28], Simple Interaction Format (SIF) and Gene Set Enrichment Analysis (GSEA) [29].

### Seamless handling of different BioPAX levels

The recently released BioPAX Level 3 introduced significant extensions to previous levels with some loss of backwards compatibility. Paxtools supports all three BioPAX levels (1, 2 and 3) and provides facilities for upgrading older BioPAX models to Level 3.

### Syntactic validation

Each operation that modifies the model is internally validated by Paxtools to comply with BioPAX syntax, including RDF well-formedness, domain and range restrictions, bidirectional links, and redundancies. This is especially useful for tools that create BioPAX such as pathway editors, converters or exporters as it allows early detection of errors, increasing the quality of the produced BioPAX data. This syntactic validation does not include more detailed checking of semantics that is performed by the BioPAX validator.

### Object/relational mapping

Paxtools comes with mappings for Java Persistence API that allows out-of-the-box persistence of BioPAX models into relational database systems, such as MySQL. This is an especially important feature for working with large biopax models, such as complete pathway database exports.

### Modular and lightweight structure

Paxtools source code is currently distributed as a modular Maven project which allows developers to easily select just the parts of Paxtools they need in their application. The Paxtools core module, which provides a complete implementation of BioPAX and read in/write out functionality, is a very compact library (400 kB) that can be expanded as required with additional modules, such as those described above.

## Results

Paxtools implements all of these features in a lightweight, extensible structure. Paxtools can be embedded in a software tool as a library, can be used for programmatic access to pathway data with Java or Jython or can be used to run most commonly used Paxtools algorithms and searches from the command line. Here, we briefly review several applications where Paxtools was used to answer biological questions or develop bioinformatics tools.

### Finding cellular processes connecting a set of genes of interest

When an experimental method identifies a set of genes associated with a phenotype, such as prostate cancer, cellular processes that connect these genes can offer a causal explanation. In this scenario, a user writes a small Java program that loads the cellular process network curated by a pathway database. Alternatively, instead of using BioPAX exports, the data can be queried dynamically from PC using the PC-client module. The program then runs a *paths-between* query, implemented in the Query package that finds the paths in the network that connect the seed genes. Entities will be mapped using UniProt identifiers and the algorithm will return the connecting sub-network in BioPAX. This BioPAX network can be visualized or used for subsequent analysis. An implementation of this scenario can be found in cBio Cancer Genomics Portal [30].

### Finding overrepresented modules or paths in a set of genomic alteration profiles

In this use case, a set of high throughput profiles are used in conjunction with pathway information to find significantly altered modules. Due to the nature of the experimental data, detailed BioPAX information, such as the phosphorylation states of interacting proteins is unnecessary in this case. To facilitate analysis, complex BioPAX pathways can be reduced to a simple interaction network using the SIF converter that effectively merges multiple states of the entity into a single node. Alteration profiles can be directly mapped onto these “entity” nodes using external references to Entrez Gene and UniProt databases as defined in BioPAX models. Existing clustering and module-finding algorithms [18], [31] can then be directly used on this SIF network. Example implementations of this usage can be found in the NetPath [32] and MeMo [33] algorithms.

### Pathway visualization and editing

Visualization is key for understanding complex relationships between biological entities. Paxtools significantly facilitates implementing Pathway visualizers and editors by handling the reading/writing and syntactic validation. Paxtools also provides additional common functionality for interactive tools such as undo/redo and object inspection. Finally, the Pathway Commons client enables pathway visualization tools to directly access and query Pathway Commons. Pathway Editors and visualizers that use Paxtools for these purposes include ChiBE [34], CellDesigner [23], Cytoscape [22] and Vanted [35].

### Protein activity network

The effect of post-translational modifications on protein activity can be useful for assessing the function of mutations in the vicinity of a modification site. Specifically, we would like to know if a given post-translational modification causes a protein to gain or lose a function. We can search pathways for processes that modify a protein and predict how this change will affect the controlled downstream processes. The algorithm also needs to consider processes that contain the protein as a part of molecular complex or as a part of a protein family. Since these inclusion relationships and the type and location of the modifications are stated explicitly in BioPAX, equivalent protein modifications can be matched to integrate findings from different pathways. A complete implementation of this algorithm can be found in the Paxtools code.

## Availability and Future Directions

Paxtools can run on any platform that has a Java Runtime Environment and was tested on most modern operating systems. Paxtools is open source and is available under the Lesser GNU public license (LGPL), which allows users to freely use the code in their software systems with a requirement for attribution. Source code for the current release (4.2.0) can be found in Software S1. A detailed manual for obtaining and using Paxtools can be found in Protocol S1. The latest sources and release bundles can be obtained from biopax.org/paxtools. Paxtools is supported by an active community of developers and users that can be reached at http://lists.sourceforge.net/lists/listinfo/biopax-paxtools. Sample code that demonstrates basic tasks and operations can be found in the org.biopax.paxtools.examples package.

A rapidly growing open-source BioPAX software infrastructure, available as part of the Pathway Commons project, is directly built on top of Paxtools, including a state-of-the-art persistence and full-text search system cPath2, an advanced validator that allows checking complex rules and best practices, a pathway alignment tool and a framework for quickly mapping experimental data on BioPAX pathways. Since these tools use a common API, it is possible to combine and re-use software components across multiple applications. Detailed examples of using Paxtools for real-life applications can be found in these projects. We encourage users to contribute to the codebase and expand it to build a pathway informatics platform that addresses the growing needs of the community.

One of our major future goals is to reach a wider community of researchers by implementing different language bindings and usage modes for Paxtools. We are planning to include bindings for the R statistical programming language and implement an interactive scripting mode for more efficient non-programmatic manipulation.

Paxtools can significantly reduce the time spent parsing, normalizing and querying BioPAX information. Without removing these barriers, it is difficult to tap into the rapidly growing computable pathway information corpus produced by multiple pathway databases and curation groups. Paxtools allows researchers to shift their effort from data plumbing to answering biological questions.

## Supporting Information

Protocol S1 Paxtools User's Guide. This guide provides details on how to obtain and use Paxtools. It also provides examples and technical details for the key features as well as a frequently asked questions section.
(PDF)

Software S1 The complete source code for all Paxtools modules as of the 4.2.0 release as a single bundle. The latest sources and release bundles can be obtained from biopax.org/paxtools.
(TAR)

## References

[1] Bader GD, Cary MP, Sander C (2006) Pathguide: a pathway resource list. Nucleic acids research 34: D504–6. 16381921 10.1093/nar/gkj126 PMC1347488

[2] Network TCGA (2008) Comprehensive genomic characterization defines human glioblastoma genes and core pathways. Nature 455: 1061–8. 18772890 10.1038/nature07385 PMC2671642

[3] Jones S, Zhang X, Parsons DW, Lin JCH, Leary RJ, et al (2008) Core signaling pathways in human pancreatic cancers revealed by global genomic analyses. Science (New York, NY) 321: 1801–6. 10.1126/science.1164368 PMC2848990 18772397

[4] Hu P, Bader G, Wigle DA, Emili A (2007) Computational prediction of cancer-gene function. Nature reviews Cancer 7: 23–34. 17167517 10.1038/nrc2036

[5] Chuang HY, Lee E, Liu YT, Lee D, Ideker T (2007) Network-based classification of breast cancer metastasis. Molecular systems biology 3: 140. 17940530 10.1038/msb4100180 PMC2063581

[6] Aerts S, Lambrechts D, Maity S, Van Loo P, Coessens B, et al (2006) Gene prioritization through genomic data fusion. Nature biotechnology 24: 537–44. 10.1038/nbt1203 16680138

[7] Chen J, Bardes EE, Aronow BJ, Jegga AG (2009) ToppGene Suite for gene list enrichment analysis and candidate gene prioritization. Nucleic acids research 37: W305–11. 19465376 10.1093/nar/gkp427 PMC2703978

[8] Vaske CJ, Benz SC, Sanborn JZ, Earl D, Szeto C, et al (2010) Inference of patient-specific pathway activities from multi-dimensional cancer genomics data using PARADIGM. Bioinformatics (Oxford, England) 26: i237–45. 10.1093/bioinformatics/btq182 PMC2881367 20529912

[9] Demir E, Cary MP, Paley S, Fukuda K, Lemer C, et al (2010) The BioPAX community standard for pathway data sharing. Nat Biotechnol 28: 935–942. 20829833 10.1038/nbt.1666 PMC3001121

[10] Yu N, Seo J, Rho K, Jang Y, Park J, et al (2012) hiPathDB: a human-integrated pathway database with facile visualization. Nucleic acids research 40: D797–802. 22123737 10.1093/nar/gkr1127 PMC3245021

[11] Sreenivasaiah PK, Rani S, Cayetano J, Arul N, Kim DH (2012) IPAVS: Integrated Pathway Resources, Analysis and Visualization System. Nucleic acids research 40: D803–8. 22140115 10.1093/nar/gkr1208 PMC3245119

[12] Cerami EG, Gross BE, Demir E, Rodchenkov I, Babur O, et al (2011) Pathway Commons, a web resource for biological pathway data. Nucleic Acids Res 39: D685–90. 21071392 10.1093/nar/gkq1039 PMC3013659

[13] Croft D, O'Kelly G, Wu G, Haw R, Gillespie M, et al (2011) Reactome: a database of reactions, pathways and biological processes. Nucleic Acids Res 39: D691–7. 21067998 10.1093/nar/gkq1018 PMC3013646

[14] Schaefer CF, Anthony K, Krupa S, Buchoff J, Day M, et al (2009) PID: the Pathway Interaction Database. Nucleic acids research 37: D674–9. 18832364 10.1093/nar/gkn653 PMC2686461

[15] Caspi R, Altman T, Dale JM, Dreher K, Fulcher CA, et al (2010) The MetaCyc database of metabolic pathways and enzymes and the BioCyc collection of pathway/genome databases. Nucleic acids research 38: D473–9. 19850718 10.1093/nar/gkp875 PMC2808959

[16] Mi H, Dong Q, Muruganujan A, Gaudet P, Lewis S, et al (2010) PANTHER version 7: improved phylogenetic trees, orthologs and collaboration with the Gene Ontology Consortium. Nucleic acids research 38: D204–10. 20015972 10.1093/nar/gkp1019 PMC2808919

[17] Hornbeck PV, Kornhauser JM, Tkachev S, Zhang B, Skrzypek E, et al (2012) PhosphoSitePlus: a comprehensive resource for investigating the structure and function of experimentally determined post-translational modifications in man and mouse. Nucleic acids research 40: D261–70. 22135298 10.1093/nar/gkr1122 PMC3245126

[18] Kirouac DC, Saez-Rodriguez J, Swantek J, Burke JM, Lauffenburger DA, et al (2012) Creating and analyzing pathway and protein interaction compendia for modelling signal transduction networks. BMC systems biology 6: 29. 22548703 10.1186/1752-0509-6-29 PMC3436686

[19] Searls DB (2005) Data integration: challenges for drug discovery. Nature reviews Drug discovery 4: 45–58. 15688072 10.1038/nrd1608

[20] Sorger PK (2005) A reductionist's systems biology: opinion. Current opinion in cell biology 17: 9–11. 15661513 10.1016/j.ceb.2004.12.012

[21] Kelder T, Conklin BR, Evelo CT, Pico AR (2010) Finding the right questions: exploratory pathway analysis to enhance biological discovery in large datasets. PLoS biology 8: 5. 10.1371/journal.pbio.1000472 PMC2930872 20824171

[22] Cline MS, Smoot M, Cerami E, Kuchinsky A, Landys N, et al (2007) Integration of biological networks and gene expression data using Cytoscape. Nature protocols 2: 2366–82. 17947979 10.1038/nprot.2007.324 PMC3685583

[23] Mi H, Muruganujan A, Demir E, Matsuoka Y, Funahashi A, et al (2011) BioPAX support in CellDesigner. Bioinformatics (Oxford, England) 27: 3437–8. 10.1093/bioinformatics/btr586 PMC3232372 22021903

[24] Li C, Donizelli M, Rodriguez N, Dharuri H, Endler L, et al (2010) BioModels Database: An enhanced, curated and annotated resource for published quantitative kinetic models. BMC systems biology 4: 92. 20587024 10.1186/1752-0509-4-92 PMC2909940

[25] Babur O, Demir E, Gönen M, Sander C, Dogrusoz U (2010) Discovering modulators of gene expression. Nucleic Acids Res 38: 5648–5656. 20466809 10.1093/nar/gkq287 PMC2943625

[26] Dogrusoz U, Cetintas A, Demir E, Babur O (2009) Algorithms for effective querying of compound graph-based pathway databases. BMC Bioinformatics 10: 376. 19917102 10.1186/1471-2105-10-376 PMC2784781

[27] Kerrien S, Orchard S, Montecchi-Palazzi L, Aranda B, Quinn AF, et al (2007) Broadening the horizon–level 2.5 of the HUPO-PSI format for molecular interactions. BMC Biol 5: 44. 17925023 10.1186/1741-7007-5-44 PMC2189715

[28] van Iersel MP, Villéger AC, Czauderna T, Boyd SE, Bergmann FT, et al (2012) Software support for SBGN maps: SBGN-ML and LibSBGN. Bioinformatics 28: 2016–21. 22581176 10.1093/bioinformatics/bts270 PMC3400951

[29] Subramanian A, Tamayo P, Mootha VK, Mukherjee S, Ebert BL, et al (2005) Gene set enrichment analysis: a knowledge-based approach for interpreting genome-wide expression profiles. Proceedings of the National Academy of Sciences of the United States of America 102: 15545–50. 16199517 10.1073/pnas.0506580102 PMC1239896

[30] Cerami E, Gao J, Dogrusoz U, Gross BE, Sumer SO, et al (2012) The cBio cancer genomics portal: an open platform for exploring multidimensional cancer genomics data. Cancer discovery 2: 401–4. 22588877 10.1158/2159-8290.CD-12-0095 PMC3956037

[31] Ma'ayan A (2009) Insights into the organization of biochemical regulatory networks using graph theory analyses. The Journal of biological chemistry 284: 5451–5. 18940806 10.1074/jbc.R800056200 PMC2645810

[32] Cerami EG, Bader GD, Gross BE, Sander C (2006) cPath: open source software for collecting, storing, and querying biological pathways. BMC bioinformatics 7: 497. 17101041 10.1186/1471-2105-7-497 PMC1660554

[33] Ciriello G, Cerami E, Sander C, Schultz N (2012) Mutual exclusivity analysis identifies oncogenic network modules. Genome research 22: 398–406. 21908773 10.1101/gr.125567.111 PMC3266046

[34] Babur O, Dogrusoz U, Demir E, Sander C (2010) ChiBE: interactive visualization and manipulation of BioPAX pathway models. Bioinformatics (Oxford, England) 26: 429–31. 10.1093/bioinformatics/btp665 PMC2815657 20007251

[35] Czauderna T, Klukas C, Schreiber F (2010) Editing, validating and translating of SBGN maps. Bioinformatics (Oxford, England) 26: 2340–1. 10.1093/bioinformatics/btq407 PMC2935428 20628075

